# Validity and reliability of performance tests as balance measures in patients with total knee arthroplasty

**DOI:** 10.1186/s43019-022-00136-4

**Published:** 2022-03-10

**Authors:** Devrim Can Sarac, Bayram Unver, Vasfi Karatosun

**Affiliations:** 1 Department of Physiotherapy and Rehabilitation, Faculty of Health Sciences, Izmir Katip Celebi University, Izmir, Turkey; 2 School of Physical Therapy and Rehabilitation, Dokuz Eylul University, Floor: 3, Balcova, Izmir, Turkey; 3 Department of Orthopedics and Traumatology, School of Medicine, Dokuz Eylul University, Balcova, Izmir, Turkey

**Keywords:** Knee arthroplasty, Balance, Performance test, Validity, Reliability

## Abstract

**Purpose:**

This study aimed to investigate validity and reliability of the Timed Up and Go Test (TUG), 10 Meter Walk Test (10MWT), Single Leg Stance Test (SLST), 2 Minute Walk Test (2MWT), and Five Times Sit-to-Stand Test (5xSST) for evaluating balance in patients with total knee arthroplasty (TKA).

**Materials and Methods:**

This cross-sectional study included 41 participants who had undergone TKA 6 months before the study due to osteoarthritis. Participants performed the TUG, 10MWT, SLST, 5xSST, and 2MWT. Each of the tests was performed twice, with a 1-day interval between tests. Intraclass correlation coefficient (ICC) models were used to determine the test–retest reliability. The level of correlations between performance tests and Berg Balance Scale and Fall Efficacy Scale-International were used to establish concurrent and convergent validity of the performance tests, respectively.

**Results:**

The mean age of the subjects was 64.07 ± 10.57 years. All tests showed excellent reliability (ICC > 0.94), excluding SLST that demonstrated good test–retest reliability (ICC = 0.72). All of the tests (SLST, 10MWT, 5xSST, 2MWT, TUG) were found to have good validity (rho > 0.704).

**Conclusions:**

According to these results, TUG, 10MWT, SLST, 5xSST, and 2MWT are reliable and valid outcome measures in patients with TKA, and could be used to assess balance after TKA surgery.

## Introduction

Total knee arthroplasty (TKA) is a surgical procedure in which an artificial joint or prosthesis replaces the damaged knee joint surfaces [1, 2]. It is one of the most frequently used surgical procedures to reduce pain and improve functional range of motion in patients with advanced knee osteoarthritis (OA). However, loss of function may persist even in the late postoperative period in many patients after TKA surgery [2, 3]. One of the most important functional impairments is the balance deficit, which increases the risk of falls in these patients [3, 4]. Although the reduction in pain and the improvements in knee functions have a positive effect on balance, replacement of the joint surfaces and postoperative muscle weakness may lead to diminished proprioception in the knee joint [3–6]. Additionally, factors such as valgus deformity, OA grade before surgery, and the presence of joint laxity also contribute to the proprioceptive deficit, which increases balance deficits and fall risk in patients after TKA [6, 7]. Thus, it is necessary to evaluate the balance after TKA surgery in a comprehensive manner, which is only possible with the use of valid and reliable tests.

Computerized measurement methods, the Berg Balance Scale (BBS), and different versions of the Balance Evaluation Systems Test (BESTest), have been used to evaluate balance after TKA in previous studies [8]. The BBS is a valid and reliable tool for assessing balance for many disease groups. However, as it was developed to measure balance in patients with neurological disorders that have severe balance impairments, BBS has low sensitivity and shows a high ceiling effect in patient populations with mild or moderate balance loss. Thus, the usability of BBS in TKA patients is very limited [9]. Chan et al. demonstrated that the BESTest and its modified versions are valid, reliable, and responsive tools for assessing the balance in patients with TKA [1, 8]. However, testing can take between 15 and 45 min, and the necessity of training to perform the tests may limit their clinical utility.

Computerized systems provide objective data with high accuracy and consistency in the evaluation of dynamic and static balance. However, the length of time it takes to perform these tests and the high cost of the necessary equipment complicate their use in the clinic and often lead computerized systems to be used only in scientific research [10]. Conversely, performance tests provide fast and objective data without requiring much equipment, hence they are used more frequently in the evaluation of balance in clinical settings.

The Timed Up and Go Test (TUG), 10 Meter Walk Test (10MWT), Single Leg Stance Test (SLST), Five Times Sit-to-Stand Test (5xSST), and 2 Minute Walk Test (2MWT), are all frequently used in assessing functional recovery and performance after TKA, and to evaluate the effectiveness of postoperative exercise regimes [11–13]. A previous study found that SLST, TUG, 5xSST, and the Six Minute Walk Test are the most common tests used by physiotherapists to evaluate functional recovery after TKA [14]. Although these tests are valid and reliable for measuring balance in different populations [15–17], there are not enough studies that examined the validity and reliability of these tests on assessing the balance in patients with TKA. Therefore, this study aimed to investigate the validity and reliability of TUG, 10MWT, SLST, 5xSST, and 2MWT in evaluating balance in patients who had undergone bilateral TKA surgery.

## Methods

### Design

This study was designed as an observational cross-sectional study. To examine the validity of the performance tests, BBS was used as a reference standard due to its widespread use in clinical and research settings [8]. The level of correlation between performance test scores and BBS scores was used for further investigation of concurrent validity, and the level of correlations between performance test scores and Fall Efficacy Scale-International (FES-I) scores were used for further investigation of convergent validity. Participants performed TUG, 10MWT, SLST, 5xSST, and 2MWT twice, with an interval of 1 day to investigate their test–retest reliability. Performance tests were performed in random order. All tests were conducted by the same assessor.

### Participants

Forty-one patients who had completed their sixth month after simultaneous primary bilateral TKA surgery due to severe knee OA were included in the study. All patients were operated on by the same surgeon (V.K.). Previous research determined that the patients demonstrate rapid functional recovery up to 6 months; however, little to no functional improvement occurs between the seventh month and one year after TKA surgery. Therefore, the seventh month after the surgery was set as the evaluation time for this study [18]. Inclusion criteria were being 40 years old or older and being able to stand and walk without the need for any auxiliary equipment. Exclusion criteria were having had a different lower extremity surgery or having neurological and/or orthopedic impairments that may affect gait and balance.

The priory sample size calculation section of the G*Power 3.1.9.2 program (Software, concept, and design of the University of Kiel, Germany, free Windows software by Franz) was used to determine a minimum number of subjects required for the study. Data from a similar previous study published by Chan et al. was used in the calculation of sample size [8]. An expected Intraclass Correlation Coefficient (ICC) of 0.90 and a null ICC of 0.75 were assumed. Type I error rate of 5% and Type II error rate of 80% revealed a minimum number of 34 subjects were required. To ensure that the study had sufficient power to determine the relationship between measurements, 20% more subjects were added to the required number, thus a total of 41 subjects were required.

The ethics committee approval of the study was obtained from the Dokuz Eylul University Ethical Committee for Non-Interventional Studies with 2618-GOA protocol number and 2016/12-34 decision number. Written informed consent was gathered from all the participants and all procedures were performed following the Declaration of Helsinki.

### Measurement tools

#### BBS

The test takes 15–30 minutes to complete and includes 14 different items. BBS begins with the activity of “standing up” and progresses up to “standing on one leg.” At the end of the test, a total score between 0 and 56 is generated by summing the scores that patients have obtained from 14 items [9]. Chan et al. showed that BBS has excellent interrater reliability (ICC = 0.98), test–retest reliability (ICC = 0.97), and internal consistency (Cronbach alpha = 0.97) in patients with TKA, however, they also reported that at 12 and 24 weeks after TKA, BBS shows a ceiling effect [8]. BBS has validity and reliability in Turkish language [19].

#### FES-I

The FES-I is a short, easy-to-use assessment tool based on self-reporting. It measures the concern of falling during different social and physical activities at home and outside, regardless of the person performing the relevant activities. The questionnaire consists of 16 items. The lowest score of 16 indicates the absence of fall anxiety, the highest score of 64 indicates high fall anxiety. The scale has validity and reliability in Turkish [20].

#### TUG

To measure the TUG time of participants, a colored tape was marked 3meters (m) away from an armless chair in which participants were sat. Participants were asked to walk 3 m, turn around the marked tape, and return to the chair as fast as they could. A timer was set as soon as the patient got up from the chair and was stopped when the patient was seated again. At least one practice trial was performed before the test [16].

#### 10MWT

A 20 m path was used for the 10MWT. The initial and final 5 m of the 20 m path were marked for acceleration and deceleration phases of the test. These additional spaces for acceleration and deceleration were outside the data collection area. Participants were asked to walk as fast as possible. The stopwatch was started when the participant’s leg crossed the 5 m acceleration mark and stopped as soon as the participant’s leg crossed the 15 m deceleration mark. Participants were given at least one trial test before the actual test [11].

#### SLST

The test was performed with the subjects’ eyes open, their hands crossed across their chest, and holding their shoulders. Subjects were asked to hold their nondominant lower extremity in 90° knee flexion, while keeping it from touching other leg or floor while they are standing on their dominant leg as long as possible. The test period began as soon as the participants lifted their nondominant leg from the ground and stopped if it touched the floor or other leg, or if they used arm movements to maintain their balance. At least one practice trial was performed before the actual test [21].

#### 2MWT

A 15 m straight corridor marked with a colored band at the start and finish points were used for the 2MWT. The participants were asked to walk between the marked points as fast as they could walk without stopping. The test started with the “start” command and stopped after 2 minutes. The distance traveled by the participants during this period was recorded. At least one practice trial was performed 30 minutes before the actual test [22].

#### 5xSST

An armless chair with 45 cm height, flat back, and a firm seat was used for the 5xSST. Subjects sat with their arms crossed across their chest and their hands holding their shoulders. In this starting position, they were asked to stand up and sit down from the chair five times as quickly as they could. A stopwatch was started when they lifted their hips from the seat and stopped at the end of the fifth repetition when they sat down for the last time. At least one practice trial was performed before the actual testing [15].

### Statistical analysis

Data analysis was performed using the Statistical Package for the Social Sciences (SPSS Inc. Version 21; IBM, Raleigh, NC, ABD) for the Windows program. The normality of the data was checked using the Shapiro–Wilk test. Continuous data were expressed as mean, standard deviation (SD), median, and interquartile range (IQR) where appropriate, and categorical data was expressed as frequency and percentile (%). The *p* values were deemed significant at < 0.05. The Spearman’s correlation coefficient (rho) was used to investigate the relationship between TUG, 10MWT, SLST, 5xSST, and 2MWT, and BBS and FES-I to establish the validity of the performance tests. Correlations were interpreted as follows: > 0.90 excellent, 0.90–0.71 good, 0.70–0.51 moderate, 0.50–0.31 fair,  ≤ 0.3 poor [23].

A two-way mixed model ICC was used to assess intrarater reliability between two different measurement times. Standard error measurement (SEM) values were calculated using the formula *“SEM* = *SD x* $\sqrt{1-ICC}"$. Minimal detectable change at 95% confidence interval (MDC_95_) values were also calculated by using the formula *“MDC*_*95*_ = *1.96* × *SEM x *$\sqrt{2}$*”* [24]. The obtained ICC results were interpreted as proposed by Bland and Altman (≤ 0.20 poor, 0.21–0.40 fair, 0.41–0.60 moderate, 0.61–0.80 good, and 0.81–1.00 excellent) [25].

## Results

The study was completed with 41 subjects. The mean age of the participants was 64.07 ± 10.57 years. The demographic characteristics (age, height, weight, body mass index, gender, occupation, and marital status) of the subjects are presented in Table 1. Results of BBS, FES-I, TUG, 10MWT, SLST, 2MWT, and 5xSST tests are presented in Table 2.

**Table 1:** Characteristics of participants

Characteristic	Mean ± SD or n (%)
Age (years)	64.07 ± 10.57
Height (cm)	156.96 ± 8.79
Weight(kg)	75.74 ± 9.65
BMI (kg/m^2^)	31.11 ± 5.39
Gender	
Female Male	34 (82.9%) 7 (17.1%)
Occupation	
Employed Unemployed	14 (34.1%) 27 (65.9%)
Marital status	
Married Single	35 (85.36%) 6 (14.64%)

*SD* standard deviation, *n* number *cm* centimeters, *kg* kilogram, *BMI* body mass index, *m* meters

**Table 2:** Performance test scores of participants

Test	Median (IQR)
BBS (score)	54.0 (50.75/55.0)
FES-I (score)	22.0 (17.75/27.25)
TUG (sec)	9.60 (7.87/12.42)
5xSST (sec)	11.46 (10.03/15.2)
SLST (sec)	10.01 (4.2/17.79)
10MWT (sec)	9.31 (7.37/11.1)
2MWT (m)	150.0 (119.25/161.25)

*IQR* interquartile range, *BBS* Berg Balance Scale, *FES-I* Fall Efficacy Scale-International, *TUG* Timed Up and Go Test, *10MWT* 10 Meter Walk Test, *SLST* Single Leg Stance Test, *2MWT* 2 Minute Walk Test, *5xSST* Five Times Sit-to-Stand Test, *sec* seconds, *cm* centimeters; *m* meters

Spearman’s correlation revealed that TUG, 10MWT, SLST, 5xSST, and 2MWT all showed a good correlation with BBS (rho = 0.749, *p* < 0.01) (Table 3). A good level of correlation was observed between 5xSST and FES-I, while TUG, 10MWT, 2MWT, and SLST demonstrated a moderate level of correlation with FES-I (0.692 ≥|rho|≥ 0.540, *p* < 0.01) (Table 3).

**Table 3:** Correlations between performance tests and BBS and FES-I

	BBS	*p*-Value*	FES-I	*p*-Value*
TUG	−0.813	< 0.01	0.692	< 0.01
5xSST	−0.765	< 0.01	0.749	< 0.01
SLST	0.704	< 0.01	−0.540	< 0.01
10MWT	−0.737	< 0.01	0.617	< 0.01
2MWT	0.819	< 0.01	−0.677	< 0.01

*p < 0.05, Spearman’s correlation coefficient (rho); *BBS* Berg Balance Scale, *FES-I* Fall Efficacy Scale-International, *TUG* Timed Up and Go Test, *5xSST* Five Times Sit-to-Stand Test, *SLST* Single Leg Stance Test, *10MWT* 10 Meter Walk Test, *2MWT* 2 Minute Walk Test

Results of the reliability analyses showed that TUG, 10MWT, 2MWT, and 5xSST had excellent test retest reliability (ICC > 0.80) and SLST had good test–retest reliability (ICC = 0.72) (Table 4). SEM and MDC_95_ values of the performance tests are presented in Table 4.

**Table 4:** Test–retest reliability, standard error measurements, and minimal detectable change values of the performance tests

	First evaluation, mean ± SD	Second evaluation, mean ± SD	ICC (95% CI)	SEM	MDC_95_
TUG (sec)	10.81 ± 4.5	10.71 ± 5.05	0.94 (.89–.97)	1.10	3.04
5xSST (sec)	13.66 ± 6.33	13.14 ± 5.09	0.96 (0.92–0.98)	1.26	3.49
SLST (sec)	13.96 ± 13.62	19.47 ± 19.44	0.72 (0.47–0.85)	7.08	19.62
10MWT (sec)	9.91 ± 3.43	9.73 ± 3.08	0.97 (0.94–0.98)	0.59	1.63
2MWT (m)	143.41 ± 36.85	144.08 ± 37.54	0.94 (0.90–0.98)	9.02	25.00

*SD* standard deviation, *ICC (95% CI)* interclass correlation coefficients in 95% confidence interval, *SEM* standard error measurement, *MDC*_*95*_ minimal detectable change in 95% confidence interval, *TUG* Timed Up and Go Test, *10MWT* 10 Meter Walk Test, *SLST* Single Leg Stance Test, *2MWT* 2 Minute Walk Test, *5xSST* Five Times Sit-to-Stand Test, *sec* seconds, *cm* centimeters, *m* meters

## Discussion

It has been reported that an 85–90% decrease in pain and a 70– 80% improvement in function is expected in patients with TKA. However, loss of proprioception due to extracted tissues and postoperative muscle weakness may lead to balance deficits [3, 4, 26]. Thus, evaluating balance with valid and reliable objective assessment tools is necessary after surgery. Heretofore, only two studies investigated the reliability of TUG, 10MWT, and 2MWT, and no studies have examined the validity of the performance tests utilized in this study for evaluating balance in TKA patients [8, 11, 22]. Although these performance tests are validated for evaluating balance in different patient populations [15–17], the calculated psychometric properties are specific to that population. Thus, validating these tests in TKA patients is requisite.

TUG is a timed performance test that evaluates balance, gait speed, and function [16]. The Spearman correlation test and ICC analyses indicated that TUG is a valid and reliable test for assessing balance after TKA. SEM and MDC_95_ values ​​were also calculated as 1.1 s and 3.4 s, respectively for TUG in this study. In a previous study, Yuksel et al. reported that test–retest reliability of TUG was excellent in patients with TKA [22] (ICC = 0.97). In the same study, the values ​​of SEM and MDC_95_ calculated for TUG were similar to the results obtained in our study (SEM = 0.82; MDC_95_ = 2.27). Unlike our study, Yüksel et al. included patients who had undergone unilateral TKA or those using walking aids, which might be the reason for the small difference between obtained values. In another study, for patients with stage 1–3 knee OA, the interrater reliability (ICC = 0.96) and test–retest reliability (ICC = 0.97) of TUG were likewise excellent [27].

The sit-to-stand activity is a core component of daily living that requires a sufficient level of lower extremity muscle strength and balance [15]. Accordingly, several different performance tests are present for evaluating sit-to-stand function, and 5xSST is one of the most frequently utilized among these [15, 29]. In a previous study, Piva et al. found a fair correlation between the 5xSST time and hip abduction strength (*r* = −0.56) and knee extension strength (*r* = −0.44) in TKA patients [28]. In addition to muscle strength, the dynamic and rapid nature of this test allows the assessment of proprioception and balance [15, 29]. The results obtained in the present study also confirm this, as 5xSST was found to have good validity and excellent reliability for assessing balance in TKA patients.

Among the investigated tests, the lowest values for concurrent validity (*r* = 0.704) and convergent validity (*r* = −0.540) were obtained for SLST. In addition, the lowest level of test–retest reliability (ICC = 0.72) was also acquired for SLST. This result is interpreted as a good level of reliability according to the reliability levels suggested by Bland and Altman [25]. However, compared with other performance tests that demonstrated excellent reliability (ICC > 0.94), this result is lower. We believe, this might have emerged as a result of the static nature of the SLST. The items of BBS and FES-I are mostly composed of dynamic activities similar to activities of daily living. TUG, 10MWT, 5xSST, and 2MWT also include activities of daily living such as sitting down, standing, walking, and turning around a point, which require dynamic balance to be performed. On the other hand, SLST is a static test and not an activity of daily living. We believe this is the primary reason for the relatively low correlation coefficients obtained for SLST. In addition, SLST times differed greatly among subjects, which resulted in a high standard deviation. As a result, the SEM value of the patients was calculated as 7.08 s and MDC_95_ was 19.62 s. These values are almost as high as the mean values that participants obtained during SLST (Table 4). Lastly, the time difference between the first and second assessments was higher for SLST (first assessment: 13.96 ± 13.62 s, second assessment: 19.47 ± 19.44 s), which is the primary reason for low ICC values obtained for SLST.

In a previous study, Unver et al. examined the test–retest reliability of 4 and 10 m walking tests in subjects who had undergone TKA surgery [11]. They found the test–retest reliability of 10MWT was excellent (ICC = 0.95) and calculated SEM and MDC_95_ durations for the 10MWT test as 4.5 and 12.7 s, respectively. In this study, the test–retest reliability of 10MWT was also found to be excellent (ICC = 0.97), but much shorter SEM (0.59 s) and MDC_95_ (1.63 s) durations were calculated. In our opinion, the reason for this dramatic difference obtained in these two studies might be due to the discrepancy between the inclusion criteria. Unver et al. included only cases that were inpatients after TKA. Accordingly, the mean walking distance of the patients in 10MWT was 47.5 ± 21.2 s whereas in our study it was 9.91 ± 3.4 s. Functional level and knee scores of patients after TKA show continuous improvement up to 6 months [18]. As patients continue to improve for a long period after surgery, different SEM and MDC_95_ values might be required depending on the timing of the test [18].

The 6 Minute Walk Test is recommended to be performed after TKA as a predictor of functional recovery [30]. However, considering that TKA is generally a surgical procedure performed in geriatric patients, it is likely that with advanced age, comorbidities such as muscle weakness, gait disturbances, and cardiopulmonary impairments limit the utility of the 6 Minute Walk Test as a balance measure [17, 22]. The 2MWT is less affected by cardiopulmonary symptoms as it has a shorter duration compared with the 6 Minute Walk Test [17]. The 2MWT has previously been shown to be highly correlated with balance measurements [31]. Thus, in the present study, it was also employed to assess its psychometric properties in assessing balance after TKA. High values obtained in the validity and reliability analyses indicate that 2MWT is valid and reliable in assessing balance following TKA.

Yuksel et al. calculated the SEM and MDC_95_ distances for 2MWT as 5.4 and 14.96 m, respectively for TKA patients [22]. The present study calculated the SEM value as 9.02 m and the MDC_95_ value as 25 m. In their study, Yuksel et al. used a 30 m corridor, while a 15 m corridor was used in the present study. Secondly, unlike Yuksel et al., patients who were assisted-device-dependent or had undergone unilateral TKA surgery were not included in our study. Thus, we believe the difference between the calculated SEM and MDC_95_ might have occurred as a result of the corridor lengths and dissimilarities between the inclusion criteria.

TUG, 10MWT, 5xSST, and 2MWT were all found to have good validity and excellent reliability, and SLST showed good reliability. Although these results indicate these tests all have acceptable psychometric properties to use in research and clinical settings, in the opinion of the authors, some of the tests still have advantages over others owing to how they are performed. The 10MWT and 2MWT both require a long corridor, which might not always be available in a clinical setting. They also require more preparation and equipment. In addition, the 2MWT takes a longer time to perform compared with other tests in this study. Therefore, we believe that TUG and 5xSST might be more suitable for clinical use as they require minimal equipment and little space.

### Study limitations

The most important limitation of the study was the lack of predictive validity of performance tests in assessing the fall risk. This could not be evaluated as a result of the cross-sectional structure of the study. Another limitation is that, although participants were allowed to perform at least one trial before every test to eliminate the learning effect, the 1 day interval between the test and retest was relatively short. Additionally, only patients who had undergone simultaneous bilateral TKA surgery were included to increase the homogeneity of the study population, and TUG, 10MWT, 5xSST, and 2MWT require both lower extremities to be performed. A previous study by Bakirhan et al. showed that patients with bilateral TKA had better dynamic balance compared with patients with unilateral TKA [32]. Hence, hypothetically, participants may have had performed differently if the study population was formed of patients with unilateral TKA. Lastly, although the study had sufficient power to validate the performance tests according to power analysis, 41 might be a relatively small sample size to avoid effects of heterogeneity.

## Conclusions

TUG, 10MWT, 5xSST, and 2MWT are all valid and reliable performance tests in assessing balance in TKA patients. Although SLST was also found to be valid and reliable, the results suggest that other performance tests were superior to SLST in evaluating balance. The calculated SEM and MDC_95_ values can be used to interpret the results obtained in clinical settings and scientific studies. Future studies should investigate other psychometric properties of performance tests such as specificity, sensitivity, and responsiveness in measuring balance after TKA.

## Data Availability

The datasets of the current study are available from the corresponding author on reasonable request.
